# Supplementary material for: Acquisition of non-olfactory encoding improves odour discrimination in olfactory cortex
Source: Nat Commun. 2024 Jul 2;15:5572. doi: 10.1038/s41467-024-49897-4 (PMC11220071; doi:10.1038/s41467-024-49897-4)
Supplement: Supplementary file 1 — Supplementary Information [file 41467_2024_49897_MOESM1_ESM.pdf]

Supplementary Information

Acquisition of non-olfactory encoding improves odour discrimination in olfactory cortex

Noel Federman[1]*†, Sebastián A. Romano[1]*†, Macarena Amigo-Duran[1,2], Lucca Salomon[1,2], Antonia Marin-Burgin[1]*

[1]Instituto de Investigación en Biomedicina de Buenos Aires (IBioBA)-CONICET-Partner Institute of the Max Planck Society; Godoy Cruz 2390, C1425FQD, Buenos Aires, Argentina.

[2]Universidad de Buenos Aires. Facultad de Ciencias Exactas y Naturales, PhD program. Buenos Aires, Argentina.

*Corresponding authors:

nfederman@ibioba-mpsp-conicet.gov.ar, sromano@ibioba-mpsp-conicet.gov.ar,

aburgin@ibioba-mpsp-conicet.gov.ar

† These authors contributed equally: Noel Federman and Sebastián A. Romano

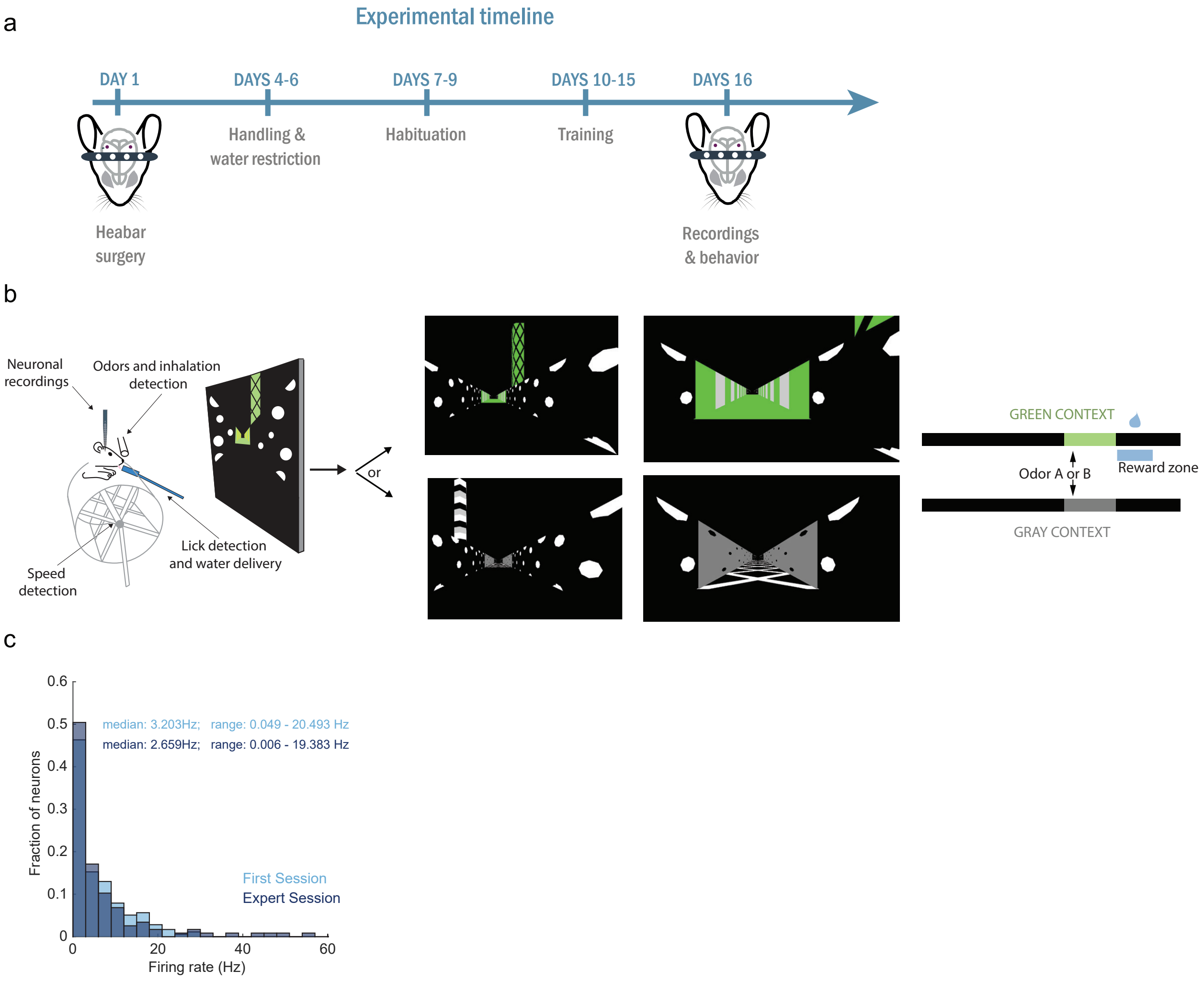

Supplementary Figure 1. Experimental setup and timeline. a, Experiment timeline indicating the training and recording protocol. **b**, Experimental setup showing the two alternative virtual reality corridors (grey or green contexts) used in the experiments. **c,** Distribution of firing rates of recorded neurons for first-session recordings (light blue) and expert-session recordings (dark blue). Source data are provided as a Source Data file.

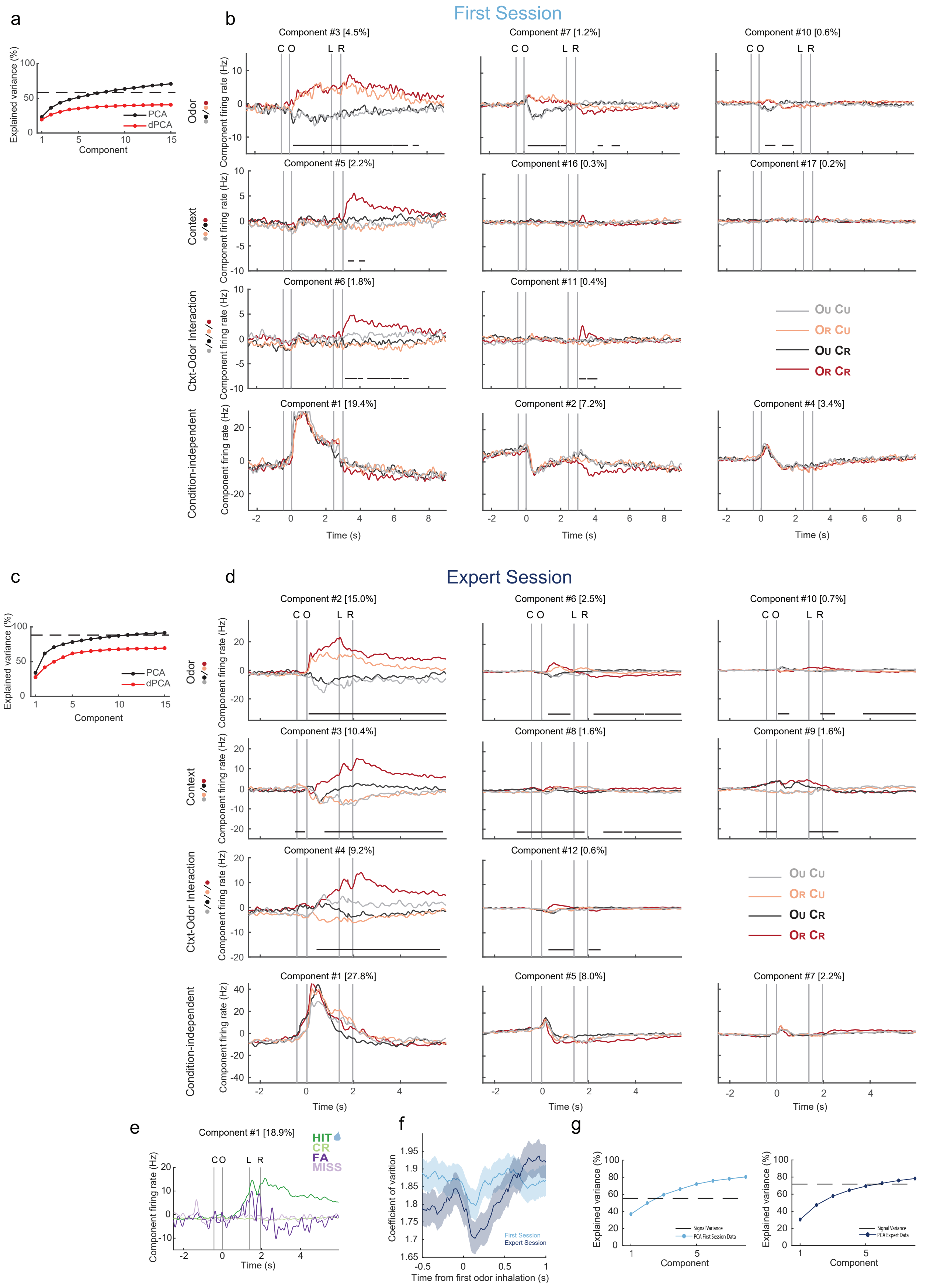

Supplementary Figure 2. Demixed PCA components. a and c, Cumulative variance explained

by dPCA and PCA from neurons recorded of animals in first session (a) or expert session (c). Dashed line shows an estimate of the fraction of signal variance in the data. **b and d,** Demixed principal components. From top to bottom panels: odour discrimination components, context discrimination components, context-odour interaction discrimination components, condition-independent components for first session (b) of expert session (d) recordings. In each subplot, the full data are projected onto the respective dPCA decoder axis. Thick black lines show time intervals during which the respective task parameters (odour, context, and context-odour interaction) can be significantly decoded from single-trial activity (see Materials and Methods). **e,** First dPCA component of piriform activity arranged according to trial outcome in expert animals (hit and correct rejection in dark and light green, false alarm and miss in dark and light purple). Variance explained by the component is shown as percentage. **f**, Coefficient of variation for odour responses aligned to first odour inhalation onset. Trial-to-trial coefficient of variation for each neuron was calculated for different odour trials and averaged. (Coefficient of variation of spike counts across trials: 1.70 +/- 0.05 for first sessions and 1.8+/-0.04 for experts, mean +/- s.e, p=0.02). **g,** Cumulative variance explained by PCA from odour responses of neurons recorded for animals in first session (top) or expert session (bottom). Odor responses comprise a time window of 1 second following first odour inhalation onset. Dashed line shows an estimate of the fraction of signal variance in the data (*i.e.*, the variance that cannot be attributed to noise variance due to variability across trials), that was higher for expert animals (71.7% and 55.4% total signal variance for experts and first sessions, respectively, matching total number of trials for both conditions). Source data are provided as a Source Data file.

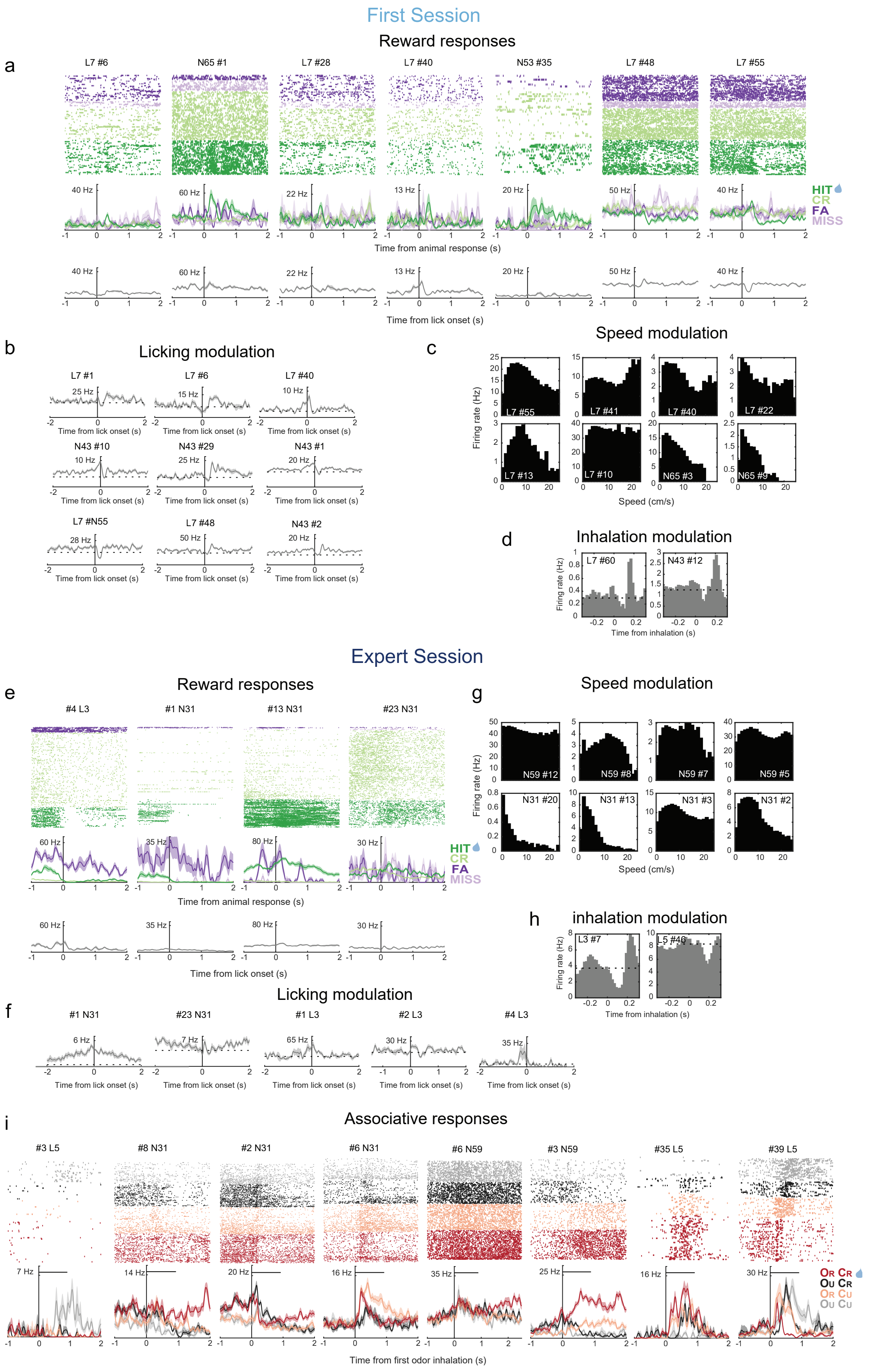

Supplementary Figure 3. a and e, Examples of reward responses are shown in raster plots of

action potentials (ticks) colour coded by trial outcome (colour labels shown on the right) for first session (a) or expert sessions (e). Middle panels show the average firing rate of each raster aligned to the animal's response after odour delivery. Bottom panels show the average firing rate of these neurons, aligned to the onset of every lick. **b and f,** Examples of responses of neurons modulated by licking in first sessions (b) or in expert sessions (f). Dashed line indicates mean firing rate. **c and g,** Examples of responses of neurons modulated by speed in first session (c) or in expert session (g). **d and h,** Examples of responses of neurons modulated by inhalation in first sessions (d) or in expert sessions (h). Dashed line indicates mean firing rate. (i) Examples of neurons with associative responses in expert animals. Horizontal black line shows odor pulse duration. Color labels shown on the right.

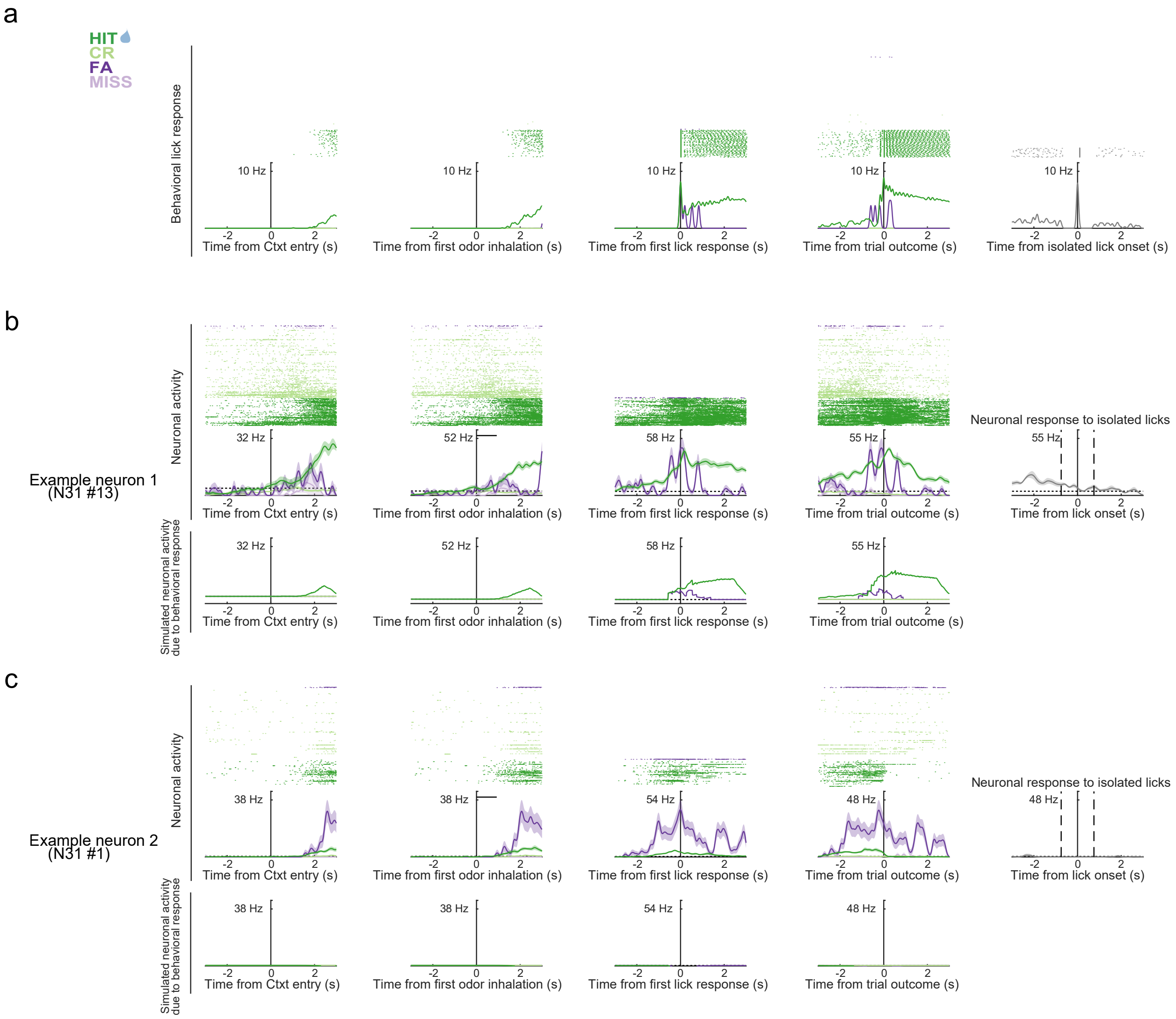

Supplementary Figure 4. Reward anticipation neurons. Differentiating pre-motor and reward anticipatory signals in single neurons. Colors indicate trial outcome: hit and correct rejection in dark and light green, false alarm and miss in dark and light purple. **a,** Behavioral lick response aligned to different trial events. Top panels shows raster plot of licks, bottom panel show instantaneous average lick rate. The column on the right shows in gray 29 licks that occurred before odor stimulation and were temporally isolated from other licks by at least 0.75s. **b,** Example of a neuron that is modulated by motor lick signals. Top panels show recorded neuronal activity aligned to different trial events, with spike raster plots on top and instantaneous average neuronal firing rates below. Horizontal dotted lines indicate mean neuronal firing rate across trials. The column on the right shows in gray the instantaneous average neuronal firing rate aligned to isolated licks. Vertical dashed lines indicate a time window between – 0.75s and 0.75s around lick onset: the neuronal firing rate curve during this time window is the estimated neuronal response to licks, fr^{licks}. Bottom panels show the simulated neuronal activity expected by the lick response. Simulations were obtained by linearly convolving fr^{licks} with the individual lick events across trials (plus a firing rate offset corresponding to the mean neuronal firing rate

across trials). Notice how simulated activity partially explains the observed instantaneous average neuronal firing rates shown on top, indicating the presence of motor modulations in this neuron. **c,** Example of a neuron with reward anticipation signals that cannot be attributed to motor activity. Notice the complete absence of neuronal firing activity around isolated licks, leading to a flat fr^{licks} that results in a simulated neuronal activity that fails to recapitulate the observed neuronal activity.

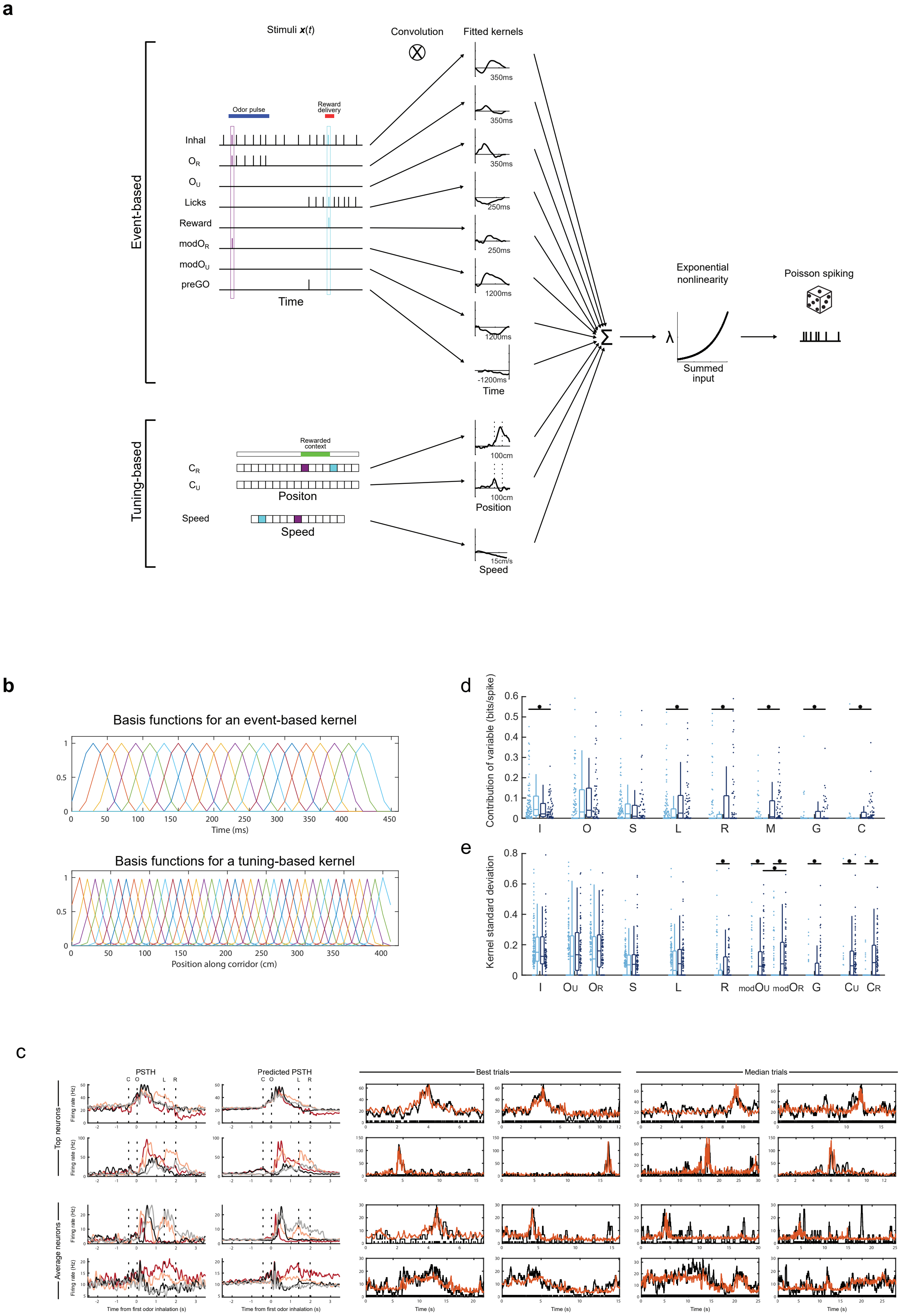
a
Stimuli x(t)
Convolution
Fitted kernels
Event-based
Odor pulse
Reward delivery
Inhal
OR
OU
Licks
Reward
modOR
modOU
preGO
Time
350ms
350ms
350ms
250ms
250ms
1200ms
1200ms
-1200ms
Time
Exponential nonlinearity
Poisson spiking
λ
Summed input
Tuning-based
Rewarded context
CR
CU
Positon
Speed
Speed
100cm
100cm
Position
15cm/s
Speed
b
Basis functions for an event-based kernel
Time (ms)
Basis functions for a tuning-based kernel
Position along corridor (cm)
d
Contribution of variable (bits/spike)
I
O
S
L
R
M
G
C
e
Kernel standard deviation
I
OU
OR
S
L
R
modOU
modOR
G
CU
CR
c
PSTH
Predicted PSTH
Best trials
Median trials
Top neurons
Average neurons
Firing rate (Hz)
Time from first odor inhalation (s)
Time (s)

Supplementary Figure 5. Parametrizations in GLM model. a, Scheme of parametrizations of task variables. Top: time evolution of variables parametrized for convolution with event-based kernels. Bottom: animal-state variables parametrized for convolution with tuning-based kernels. The instantaneous variable values in two individual 10-ms time bins are labelled in purple and cyan. **b,** Scheme of basic functions used to parametrize kernels: an example for an event-based kernel (top panel; inhalation kernel) and an example for a tuning-based kernel (bottom panel; spatial context kernel). **c**, Model-based PSTH and single-trial predictions. Examples of four neurons are shown (two "top neurons" and two "average neurons", according to PSTH prediction accuracy). Each row shows the neuron's measured and predicted PSTHs (first two columns) along with the measured and predicted single trials (last four columns, two "best trials" and two "median trials" according to trial prediction accuracy). For single trials, black ticks at the bottom indicate the observed spike train and the black trace shows an estimate of spike rate obtained by smoothing the spike train with a 300-ms boxcar, while the orange trace shows the model-based spike rate prediction for that trial. **d,** Contribution of each task variable to the GLM encoding model (bits per spike) in first and expert sessions. Black dots indicate statistically significant differences between first or expert session recordings (Wilcoxon rank sum test. I, p-value = 2.3×10^{-2}; L, p-value = 2.6×10^{-2}; R, p-value = 3.8×10^{-2}; M, p-value = 6.9×10^{-8}; G, p-value = 1.6×10^{-4}; C, p-value = 8.12×10^{-18}) **e,** Standard deviation of kernels as a function of each variable in first and expert sessions. Asterisks indicate statistically significant differences. Wilcoxon rank sum test was used for first session vs. expert session comparisons (top asterisks: R, p-value = 4.5×10^{-2}; $ModO_U$, p-value = 2.7×10^{-7}; $ModO_R$, p-value = 6.1×10^{-8}; G, p-value = 1.1×10^{-4}; C_U, p-value = 6.4×10^{-18}; C_R, p-value = 5.2×10^{-18}) and Wilcoxon signed rank paired test was used for comparison across kernel categories of each neuron (bottom asterisks: $modO_U$ vs $modO_R$,p-value = 2.6×10^{-2}). Source data are provided as a Source Data file.

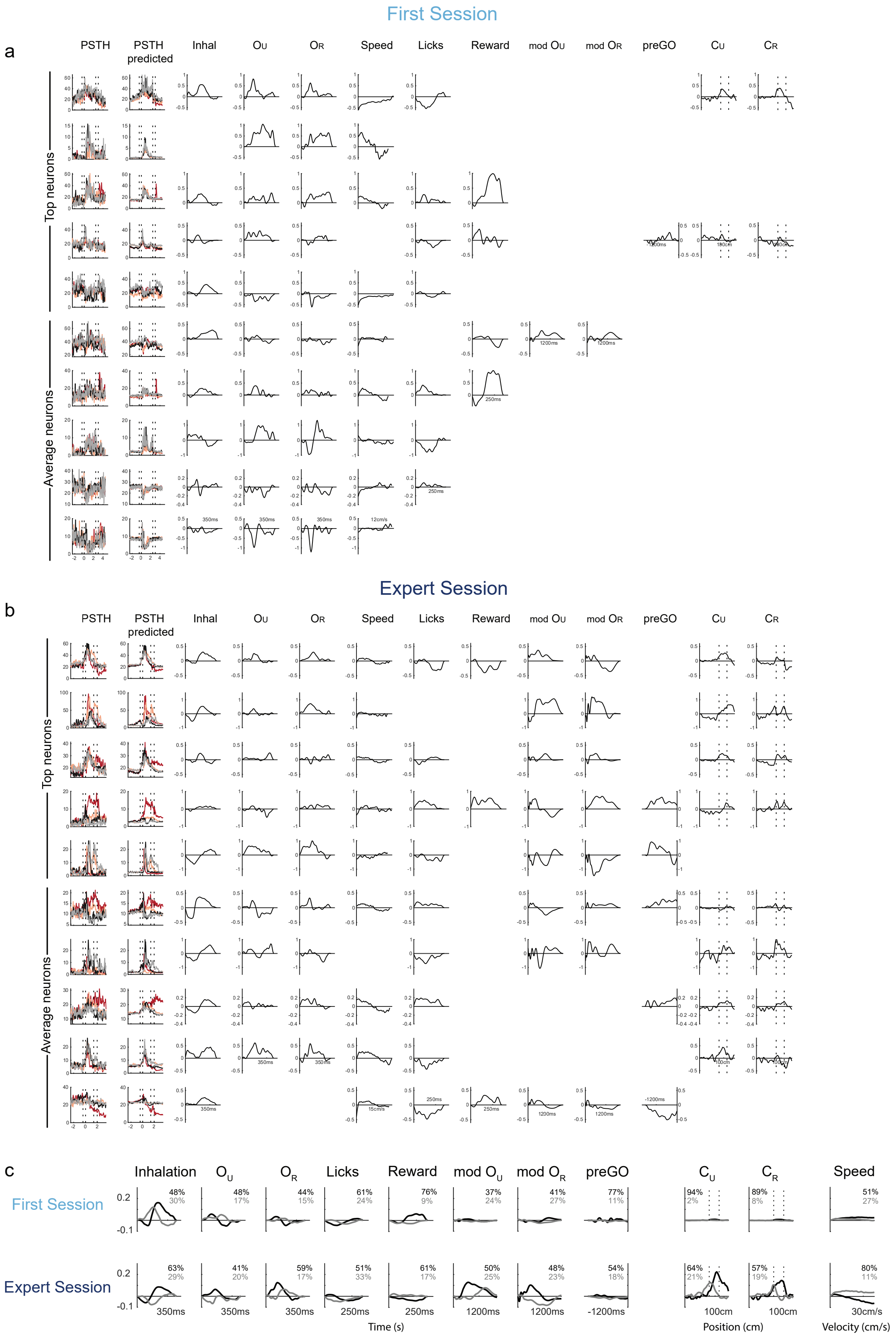

Supplementary Figure 6. Examples of kernels obtained with the GLM encoding models of

neurons. a and b, Examples of PSTHs and model-predicted PSTHs of several neurons recorded during first session (a) and during expert session (b). Panels on the right show the corresponding kernels obtained for each neuron. **c**, First (black) and second (grey) PCA component of pooled kernels for inhalation, unrewarded odour (O_U), rewarded odour (O_R), licking, reward, modulation of context (mod) onto O_U, mod O_R, activity before a GO decision (preGO), unrewarded context (C_U), rewarded context (C_R) and animal speed. Explained variance by each component is indicated in percentages, first-session recordings (top) and expert-session recordings (bottom). Parametrizations in GLM model.

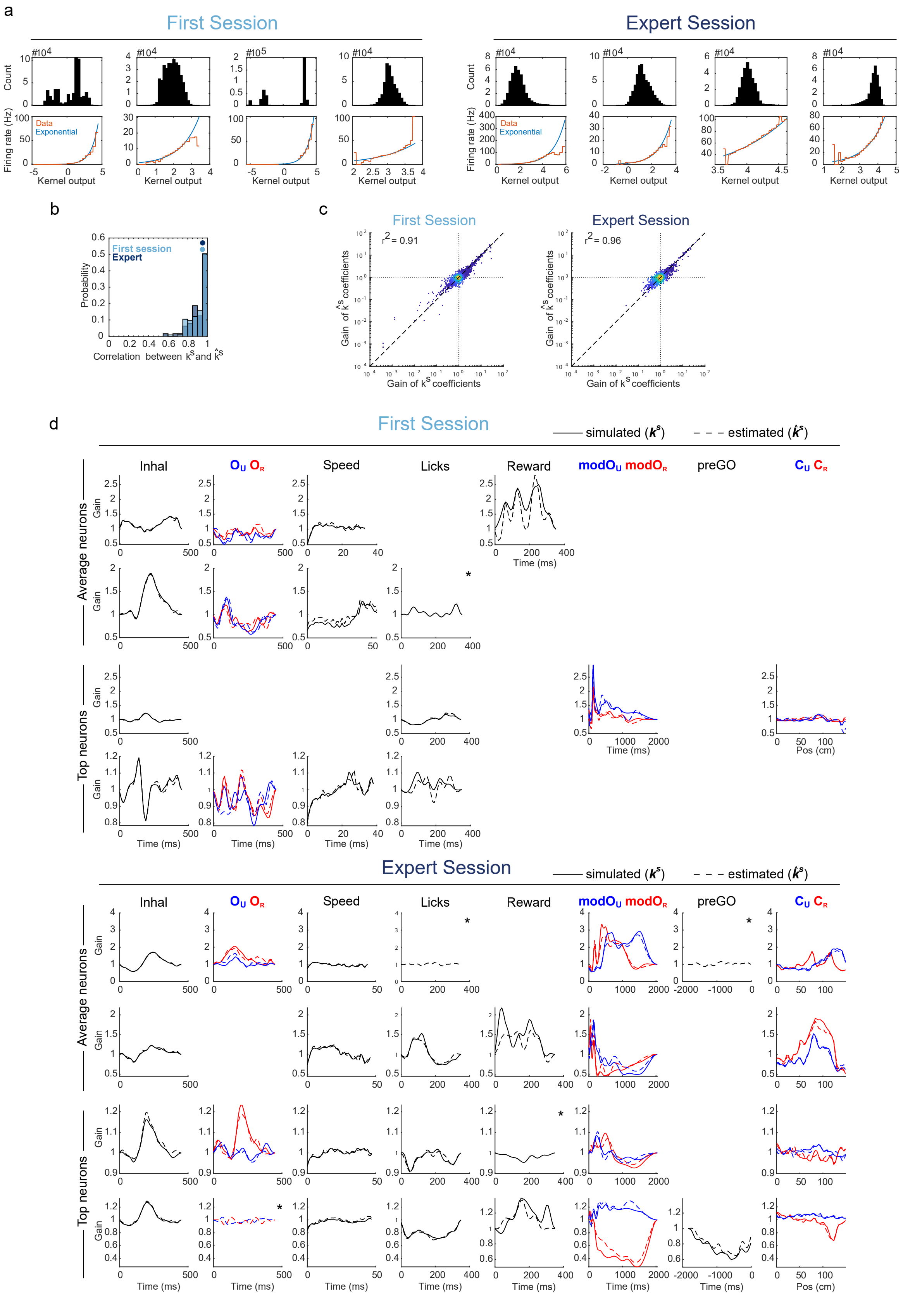

Supplementary Figure 7. Evaluation of the accuracy of GLM models. a, Validation of GLM

exponential nonlinearity. Data for four typical neurons of first session (left) and expert session (right) recordings are shown. Top panels: histograms of kernel output (i.e., $\boldsymbol{k} \otimes \boldsymbol{x}$) values for each neuron. Bottom panels: the observed spike rate for each of the kernel output bins (orange) and the predicted obtained by applying an exponential non-linearity to the kernel output bins (blue). **b-d**, Effectiveness of GLM model in quantifying the contributions of variables to the neuronal response (see *Methods*). **b**, Histogram of Pearson correlation coefficients between simulated kernels ($\boldsymbol{k}^{s}$) and estimated kernels ($\hat{\boldsymbol{k}}^{s}$) for all neurons simulated according to the recorded task data in first session and expert session animals. Dots indicate median correlation values (0.95 and 0.951 for first session and expert session, respectively). **c**, The relation between the multiplicative neuronal gain of individual $\boldsymbol{k}^{s}$ and $\hat{\boldsymbol{k}}^{s}$ coefficients. Color code indicates density of data. Diagonal dashed line indicates one-to-one relation. Vertical and horizontal dotted lines indicate a gain coefficient of 1 for $\boldsymbol{k}^{s}$ and $\hat{\boldsymbol{k}}^{s}$. Data for first and expert session are shown on the left and right panels, respectively. Coefficients of determination (r^2) are indicated. **d**, Examples of simulated $\boldsymbol{k}^{s}$ (solid lines) and estimated $\hat{\boldsymbol{k}}^{s}$ (dashed lines) kernels for average (median correlation between $\boldsymbol{k}^{s}$ and $\hat{\boldsymbol{k}}^{s}$) and top (highest correlation between $\boldsymbol{k}^{s}$ and $\hat{\boldsymbol{k}}^{s}$) neurons. Asteriks mark discrepancies in which kernels were not detected (i.e., included in $\boldsymbol{k}^{s}$ simulations but missing in $\hat{\boldsymbol{k}}^{s}$) or erroneously estimated (i.e., not included in $\boldsymbol{k}^{s}$ simulation but estimated in $\hat{\boldsymbol{k}}^{s}$), which were typically for kernels of multiplicative gain ~1 which have negligible impact on neuronal response encoding.

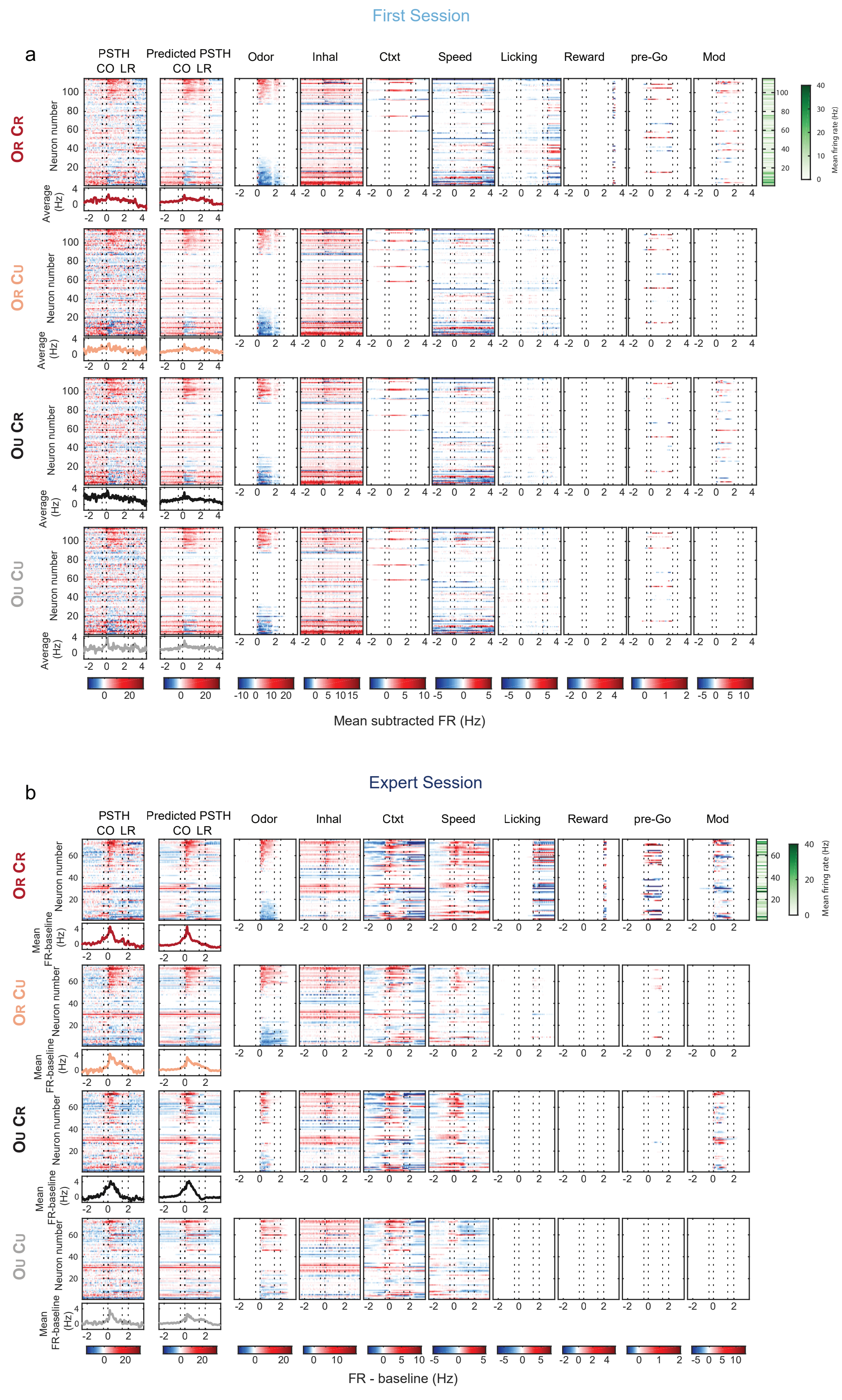

Supplementary Figure 8. Firing-rate modulations induced by individual model variables. a,

PSTHs and model-predicted PSTHs of all neurons with fitted kernels, recorded in first sessions. Panels on the right show predicted firing rate modulations (around the neuron's mean firing rate) induced by each variable (i.e., for GLM models including only that task variable). Neurons were sorted according to their average responses to ORCR trials. **b,** Same as a but for expert sessions. Source data are provided as a Source Data file.

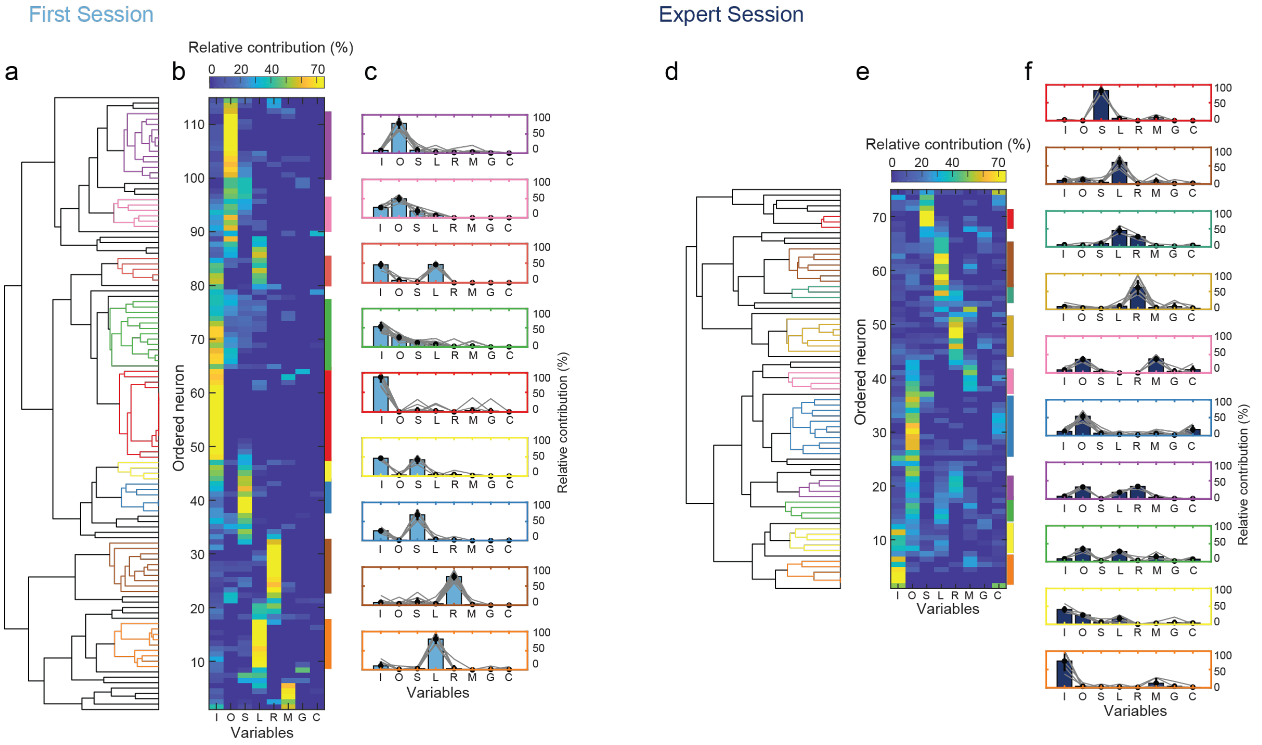

Supplementary Figure 9. Clustering neurons according to the relative contribution of their encoded task variables. a and d, Dendrograms for the hierarchical tree of first-session (a) and expert-session (d) neurons sorted according to the relative contribution of their encoded task variables. **b and e,** Relative contributions of task variables to first-session (b) and expert-session (e) neurons, sorted according to the hierarchical tree. Colour bars on the right indicate each individual cluster obtained (same colour code applies to dendrograms). **c and f,** Bar plot for each individual first-session (c) or expert-session (f) cluster, showing the relative contribution of the variables encoded by the neurons in the cluster (same data shown in Fig. 4e). Source data are provided as a Source Data file.

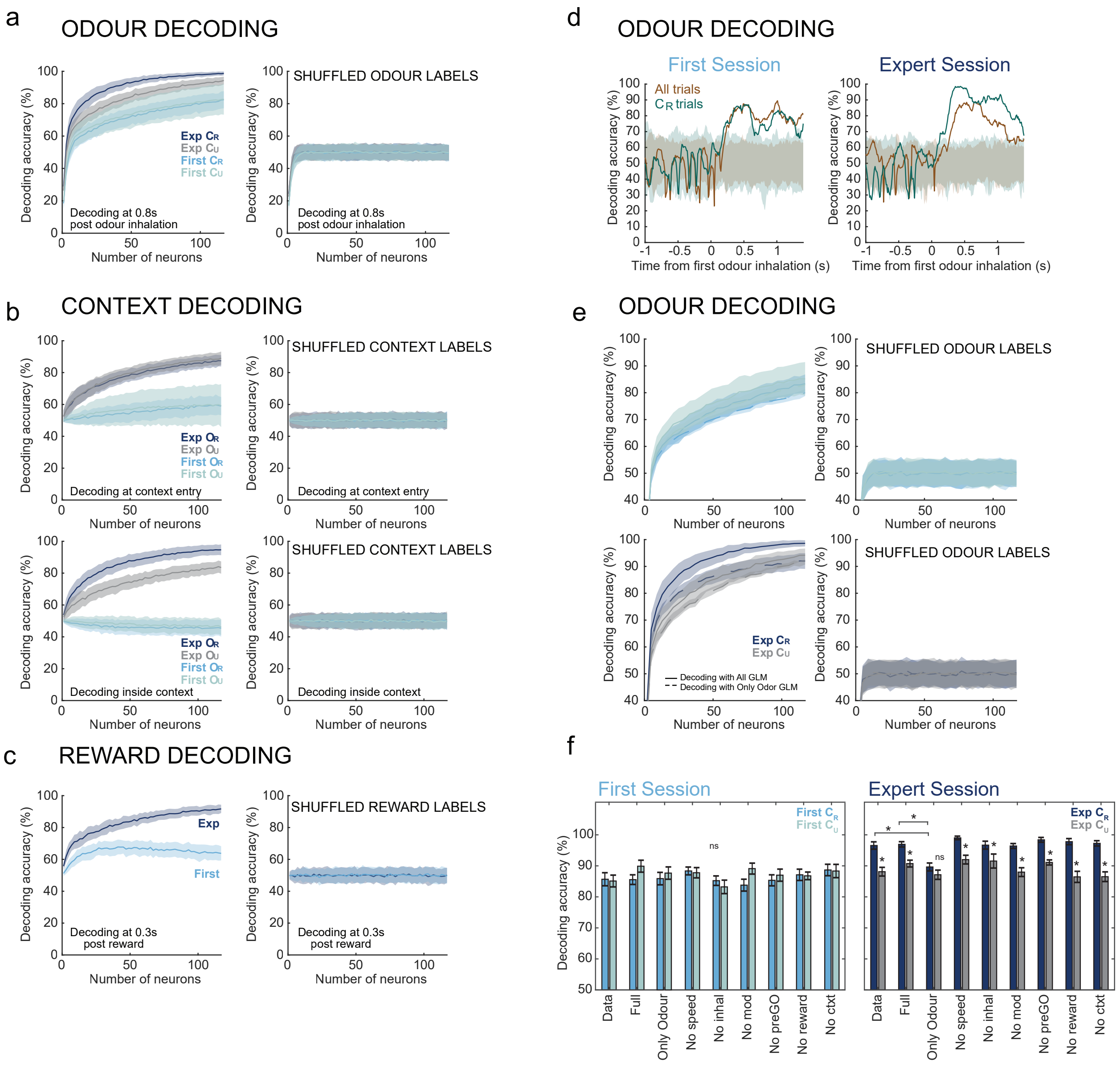

Supplementary Figure 10. **Multidimensional decoding in piriform neurons. a-c,** GLM-based trial-by-trial decoding as a function of neuronal population size, using first-session (First) and expert-session (Exp) recordings, and after shuffling trial labels. **a,** Odour identity decoding at 0.8s after first odorant inhalation in rewarded and unrewarded context trials (C_R and C_U). **b,** Context identity decoding in rewarded and unrewarded odour trials (O_R and O_U). Top, decoding at the moment of context entry. Bottom, decoding when mouse is inside context zone. **c,** Decoding reward consumption 0.3s after reward delivery. **d,** Cross-validated time-dependent odour identity decoding accuracies of a linear classifier given by the leading odour component of dPCA applied to piriform activity, using either all trials (brown line) or C_R trials only (green line). Shaded regions show distribution of decoding accuracies expected by chance as estimated by 100 iterations of a trial shuffling procedure. Statistically significant decoding is obtained

when the accuracy exceeds the accuracy by chance. First-session and expert-session data are shown on the left and right panels, respectively. **e,** Same GLM-based decoding analysis as in a, but decoding recorded data using GLM models including all fitted kernels (All GLM) and after removing contributions of non-odour-related kernels from the simulations of odour-responsive neurons (Only Odour GLM). Top, decoding accuracy for first-session data in C_R and C_U trials. Bottom, same as Top but for expert-session. Right panels show results after shuffling odour labels. For all decoding analysis shown in the figure the total number of trials used for training and testing GLM-based decoders was matched across the different trial types compared. **f,** Decoding accuracy of linear classifiers for odour identity from data (Data) and GLM model simulations, in rewarded and unrewarded context trials (C_R and C_U, respectively), for first-session (First) and expert (Exp) animals. Odor identity was decoded with the activity of a population of 100 neurons, during a 0.5s window after first odorant inhalation. Simulations were obtained for GLM models including all fitted kernels (All GLM), GLM models where we removed from odour-responsive neurons all the non-olfactory kernels (Only Odour), and GLM models where we removed from odour-responsive neurons the following single kernels: context (No Ctxt), speed (No Speed), inhalation (No Inhal), modulation of odour responses by rewarded context (No Mod), anticipation of GO response (no PreGO) and reward consumption (No Reward). 2-Way Anova, for context trial type (C_R and C_U; 1 degree of freedom), Data type (Data and simulations from the 8 GLM model types; 8 degrees of freedom). Context-Data type interaction Prob>F: 2x10-05. One-way ANOVA corrected for multiple comparisons: Data CR vs Full CR, n.s.; Data C_U vs Full C_U, n.s.; Full CR vs Only Odor CR, p-value=2x10-06; Full C_U vs Only Odor C_U, n.s.; Full CR vs every GLM simulation with single kernels removed CR, n.s.; Full CU vs every GLM simulation with single kernels removed CU, n.s.; Data CR vs Data C_U, p-value=7×10^{-07}; Full C_R vs Full C_U, p-value=1×10^{-04}; No Ctxt C_R vs No Ctxt C_U, p-value=7×10^{-07}; No Speed C_R vs No Speed C_U, p-value=7×10^{-06}; No Inhal C_R vs No Inhal C_U, p-value=7×10^{-03}; No Mod C_R vs No Mod C_U, p-value=7×10^{-07}; No PreGO CR vs No PreGO C_U, p-value=2×10^{-06}; No Reward C_R vs No Reward CU, p-value=7×10^{-07}; Only Odor CR vs Only Odour C_U, n.s. Source data are provided as a Source Data file.

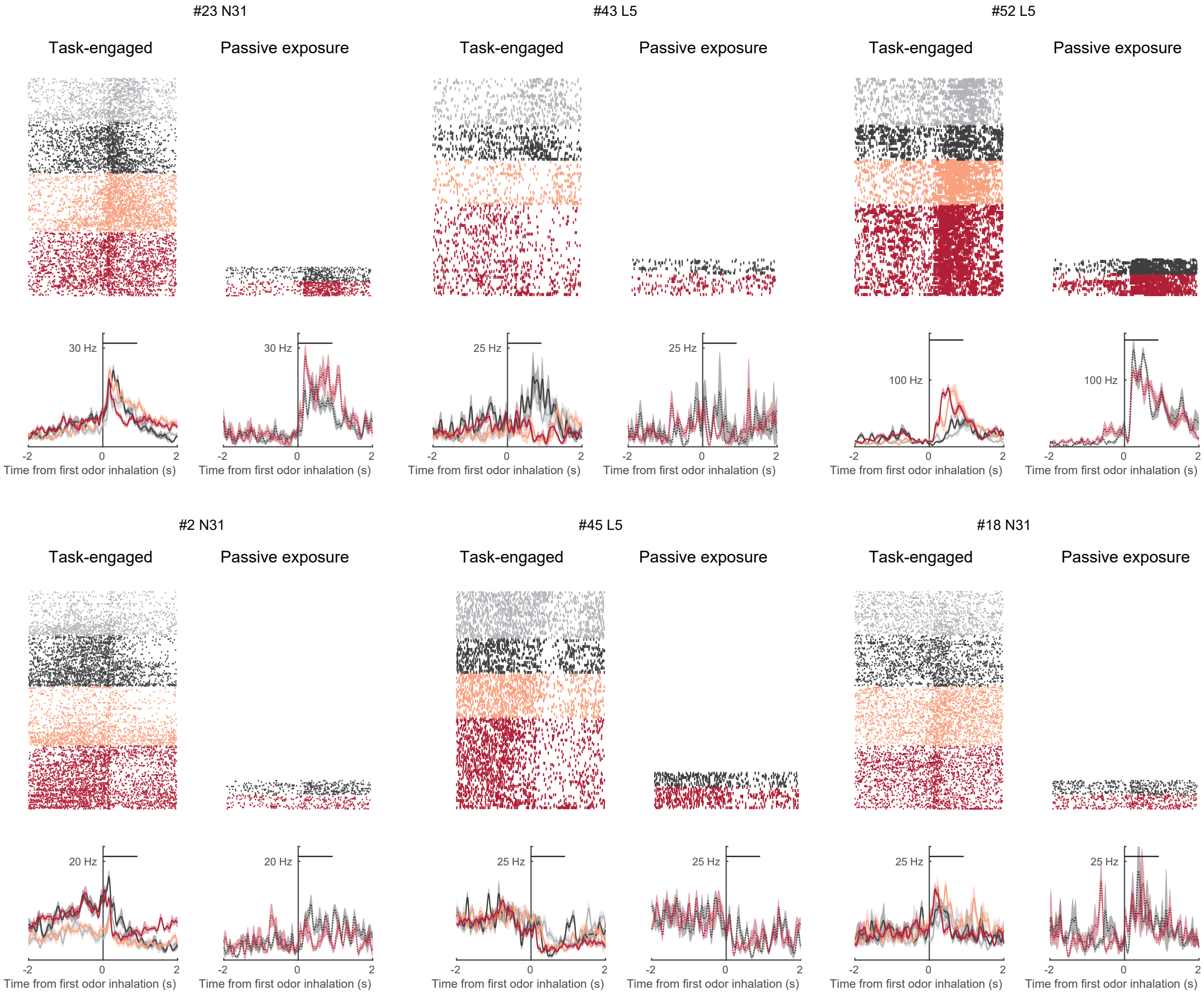

Supplementary Figure 11. Passive vs task-engaged PCx responses. Example of neuronal recordings during task engagement or when the virtual reality was turn off and animals were passively exposed to odours.

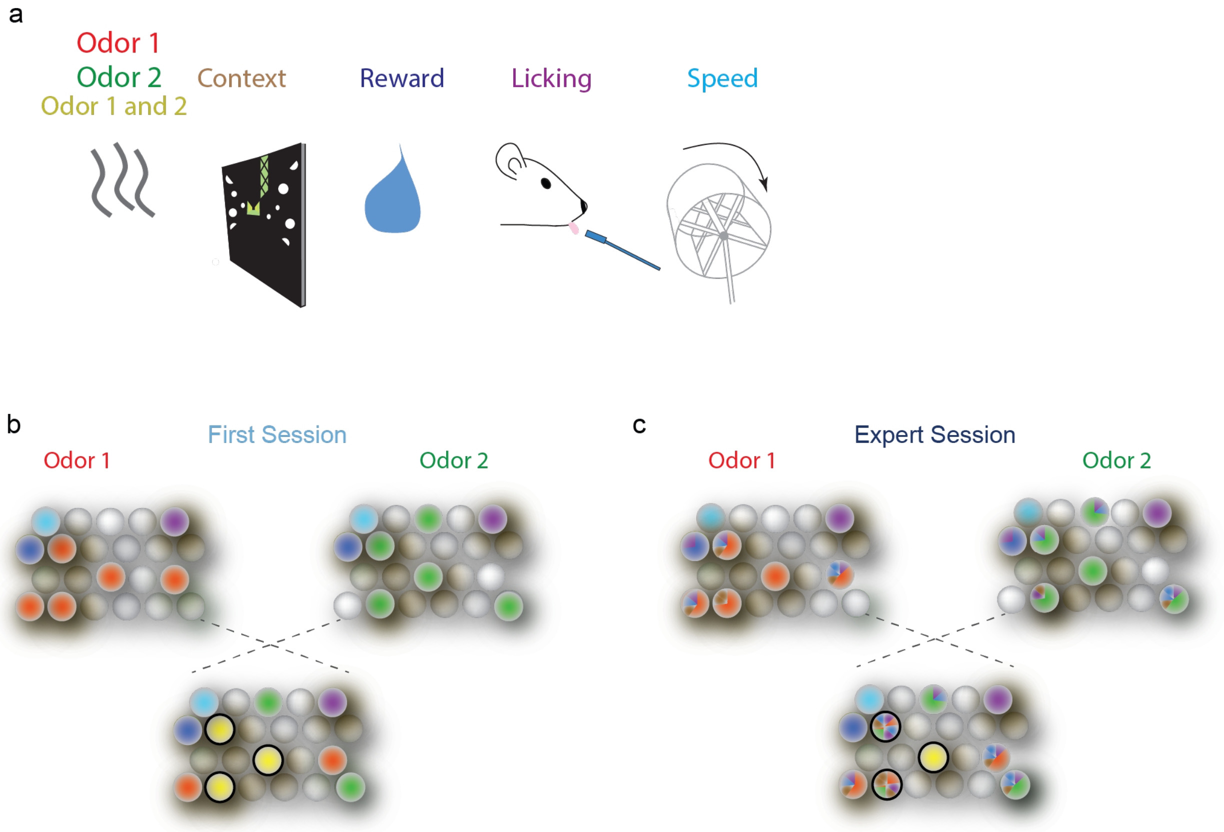

Supplementary Figure 12. Learning induces mixed-selectivity in PCx neurons that improves discrimination. a, Variables associated with the odour-visual context-reward associative task. The variables are colour coded. **b**, The scheme represents responses of neurons to different variables from a, in an animal in the first session of training. Neurons tend to respond to single variables and do not show contextual modulation. Responses to individual odours have a percentage of overlapping illustrated by the yellow neurons that do not discriminate. **c,** The scheme represents responses of neurons to different variables from a, in an animal in the expert session of training. Neurons tend to have associative responses, with a contextual modulation. Notice that the overlap of the responses to the two odours decreases due to the mixed-selectivity that arises with learning the task.
